# Arteria Lusoria With Right-Sided Aortic Arch and Atrial Septal Defect Associated With Extensive Thrombosis and Paradoxical Embolism: A Case Report

**DOI:** 10.7759/cureus.26173

**Published:** 2022-06-21

**Authors:** Tanuj Gupta, Nader Jamaleddine, Samy I. McFarlane

**Affiliations:** 1 Internal Medicine, State University of New York (SUNY) Downstate Medical Center, Brooklyn, USA

**Keywords:** paradoxical embolism, systemic thrombosis, atrial septal defect, right-sided aortic arch, arteria lusoria, aberrant right subclavian artery (arsa)

## Abstract

In this report, we present an incidental finding of a rare combination of an Aberrant Right Subclavian Artery (ARSA), or arteria lusoria, with right-sided aortic arch and atrial septal defect associated with extensive thrombosis and paradoxical embolism causing acute stroke in an octogenarian woman with COPD presenting with acute hypercapnic respiratory failure. We also discuss the various surgical approaches for management and conservative treatment alternatives in non-surgical candidates (as in this case). We believe that this is the first reported case of these combined rare anomalies in an asymptomatic patient to the best of our knowledge.

## Introduction

Aberrant right subclavian artery (ARSA), or arteria lusoria, is a developmental anomaly of the aorta in a rare subset of the general population at a suspected incidence of 0.4-1.8% [1]. ARSA refers to the origin of a right subclavian artery distal to the origin of the left subclavian artery from the aortic arch [1]. Due to this distal origin, the right subclavian artery often courses behind the esophagus to reach the right arm [1]. Although individuals are often asymptomatic (90-93%), they can present with dysphagia, chest pain, and chronic cough [2]. Arteria lusoria can also be associated with other cardiac defects, including septal defects (28%) and a right-sided aortic arch (9.2%) [2,3]. Prior literature describing an aberrant right subclavian artery with a left aortic arch and an aberrant left subclavian artery with a right aortic arch is well documented [4]. However, a literature review demonstrates that cases of an aberrant right subclavian artery with a right aortic arch are comparatively rare, especially in the setting of a concurrent septal defect. We are reporting a case of an asymptomatic aberrant retro esophageal right subclavian artery with a right-sided aortic arch and atrial septal defect in an individual with systemic thrombosis and paradoxical embolism. This constellation of findings has not been previously described.

## Case presentation

An 83-year-old female with a past medical history of Diabetes Mellitus, Hypertension, Hyperlipidemia, Chronic Kidney Disease Stage 3, COPD (dependent on supplemental oxygen), left eye blindness, and Alzheimer's Dementia was brought to the Emergency Department for lethargy and altered mental status. On presentation, the patient was in Acute Hypercapnic and Hypoxic Respiratory Failure. The patient was emergently intubated and placed on ventilator support. Given elevated risk and concern for Pulmonary Embolism (PE), CTA Chest was performed and revealed large bilateral submassive pulmonary embolisms with acute right ventricular dilatation and acute emboli in the right common carotid artery and bilateral subclavian arteries. Given alteration in mental status and inability to assess for focal deficits in addition to concerning CTA Chest findings, CT Head was performed and demonstrated extensive right-sided acute infarct involving the temporal lobe, insular cortex, occipital lobe, thalamus, and posterior limb of the internal capsule without intracranial hemorrhage or midline shift. A routine echocardiogram demonstrated significant RV dilatation with RV free wall hypokinesis/akinesis with hypercontractility of the apex, suggestive of McConnell's sign, normal LV systolic function, and upon bubble study, confirmed the presence of an atrial septal defect (ASD). Arterial Doppler ultrasound of bilateral upper extremities was performed. It showed diminished monophasic flow in the right subclavian, axillary, and brachial arteries and absent flow in the radial and ulnar arteries right subclavian artery occlusion. Venous Doppler ultrasound of the lower extremities revealed an acute DVT in the left popliteal vein.

**Figure 1:**
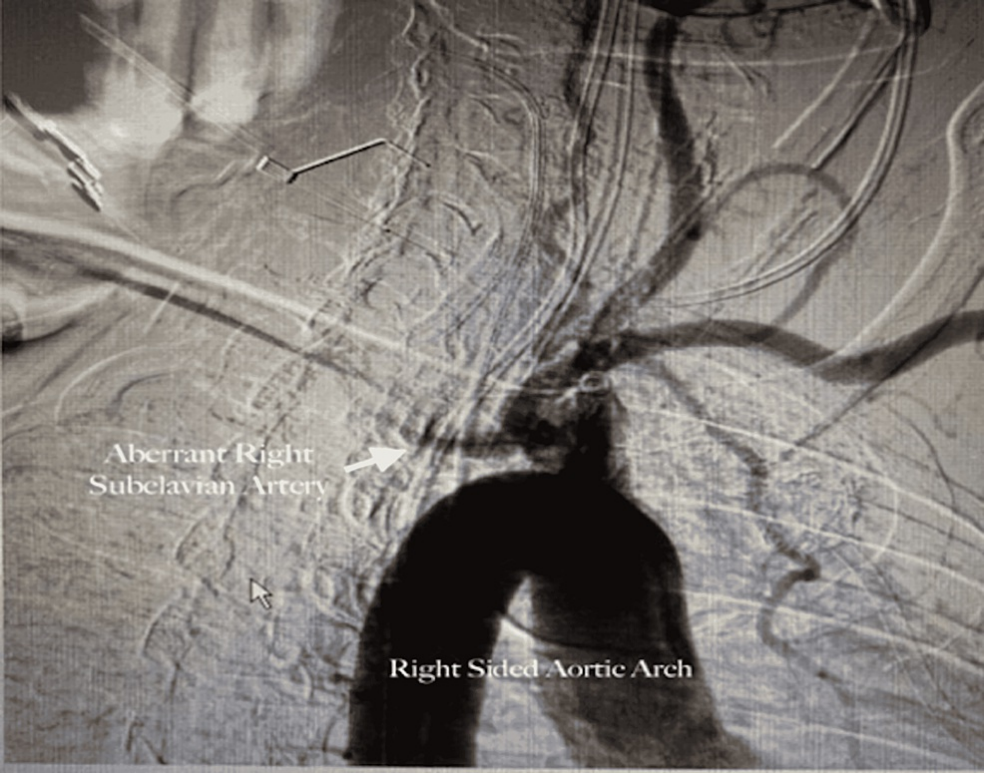
Aortography Demonstrating Occlusion of the Aberrant Right Subclavian Artery Aberrant Right Subclavian Artery with Arrow pointing to occluded Right Subclavian Artery Right Sided Aortic Arch on Aortography

**Figure 2:**
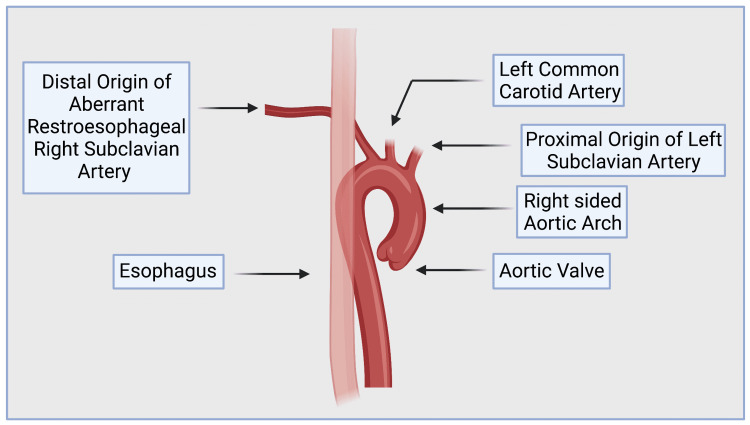
Illustration of the Distal Origin of the Aberrant Right Subclavian Artery with a Retroesophageal Course. Created on BioRender.com. Created on BioRender.com. BioRender.com provides publication and Licensing Rights.

Hyper-coagulability panel was negative, and the patient did not have an identifiable etiology for her extensive systemic thrombosis. The patient was evaluated by Neurology and deemed not suitable for acute stroke intervention or anticoagulation and was therefore taken for Inferior Vena Cava (IVC) filter placement and possible peripheral thrombectomy. Using right femoral arterial and venous access, concurrent Aortogram and IVC filter placement were performed. The Aortogram demonstrated a right-sided aortic arch with total occlusion of the brachiocephalic artery, and patent left subclavian and left common carotid arteries (Figure 1, 2). Hemostasis was achieved with an Angioseal closure device. The patient was admitted to ICU, and Cardiothoracic and Vascular Surgery were consulted for the embolic obstruction of the brachiocephalic artery and both right subclavian and right common carotid artery. She was deemed not suitable for embolectomy due to the extensive stroke and overall poor prognosis. Her ICU course was complicated by the development of cerebral edema requiring mannitol infusion and hypertonic saline, septic shock and hypotension necessitating vasopressor support, and development of Atrial Fibrillation with a Rapid Ventricular Rate necessitating Amiodarone infusion and acute kidney injury. After a prolonged hospital stay, the patient remained ventilator dependent with poor mental status, and in concordance with her family's wishes, she underwent tracheostomy and PEG placement. She was discharged to a long-term care facility.

## Discussion

During normal embryogenesis, the right fourth aortic arch regresses, forming the proximal segment of the right subclavian artery [1]. The left fourth aortic arch persists to form the medial segment of the developed aortic arch [1]. Anomalous regression of this physiological process can result in the persistence of the right aortic arch, giving rise to a distal origin of the right subclavian artery from the medial segment of the aortic arch, also known as arteria lusoria [1]. Commonly, arteria lusoria courses behind the esophagus to reach the right arm. Consequently, it may produce symptoms of dysphagia, dyspnea, chest pain, or chronic cough in a minority of patients (7-10%) [1,2]. Additionally, ARSA may be associated with the presence of other vascular anomalies such as septal defects (28%), truncus bicaroticus (19.2%), Kommerell's diverticulum (aneurysmal dilation of the descending aorta at the origin of an aberrant subclavian artery) (14.9%), aneurysms (12.8%), and right-sided aortic arches (9.2%) [2,3]. In our patient, immobility-induced venous, pulmonary, arterial, and paradoxical systemic thrombosis and embolism led to the incidental discovery of arteria lusoria, a right-sided aortic arch, and atrial septal defect.

In a study of 11,000 pathologic specimens chosen from the Registry of Cardiovascular Disease in St. Paul, Minnesota, Hugo Zapata identified 128 cases of arteria lusoria and reported that 28% of these cases had septal defects, a minority of which were atrial in origin [5,6]. The significance of this correlation arises from the controversy over the favored surgical approach for the management of symptomatic patients. Historically, the left thoracotomy has been the standard surgical approach; however, it can be technically challenging, especially when fixating the right subclavian artery and graft from the posterior to the anterior mediastinum [7]. Likewise, a right posterolateral thoracotomy approach to re-implant the right subclavian artery onto the ascending arch can lead to persistent dysphagia if the right subclavian artery is not appropriately divided close to its origin [8]. Consequently, more recent literature seems to favor the approach of a median sternotomy (vertical inline incision of the sternum) over the performance of a left or right posterolateral thoracotomy due to its improved success rates and lower risk of complications [8]. For instance, in a prior case report of a two-year-old child who presented with arteria lusoria and an ostium Secundum atrial septal defect, a median sternotomy approach, as opposed to a left thoracotomy or right supraclavicular incision, was used [2]. Another case report by Rathnakar and colleagues, in which a four-year-old girl was found to have an ARSA and a large ostium Secundum atrial septal defect, further supported a median sternotomy approach due to improved visualization of the original and new implantation sites. This approach also allows for the possible initiation of cardiopulmonary bypass, which may be imperative if uncontrollable or unexpected bleeding occurs [9,10]. In our case, these studies also highlight the benefit of early surgical intervention not only ensuring patients do not develop symptomatology from ARSA but also preventing complications of the associated vascular anomalies, such as paradoxical embolism.

## Conclusions

Although ARSA remains clinically silent in many patients, it can be associated with other vascular anomalies from which complications may arise. Our case highlights the importance of recognizing the ARSA -associated anomalies of the atrial septal defect and a right-sided aortic arch and the benefits of early surgical intervention for the simultaneous repair of ARSA and atrial septal defects in preventing paradoxical embolism.
